# Proof of Principle of Combining Fluorescence-Guided Surgery with Photoimmunotherapy to Improve the Outcome of Pancreatic Cancer Therapy in an Orthotopic Mouse Model

**DOI:** 10.1245/s10434-022-12466-4

**Published:** 2022-09-04

**Authors:** Hiroto Nishino, Michael A. Turner, Siamak Amirfakhri, Thinzar M. Lwin, Mojgan Hosseini, Bernhard B. Singer, Robert M. Hoffman, Michael Bouvet

**Affiliations:** 1 Department of Surgery, UCSD Moores Cancer Center, University of California San Diego, San Diego, CA USA; 2 Department of Surgery, VA San Diego Healthcare System, San Diego, CA USA; 3 Department of Surgery, Graduate School of Medicine, Kyoto University, Kyoto, Japan; 4 Department of Surgical Oncology, Dana Farber Cancer Center, Boston, MA USA; 5 Department of Pathology, University of California San Diego, San Diego, CA USA; 6 Medical Faculty, Institute of Anatomy, University of Duisburg-Essen, Essen, Germany; 7 AntiCancer, Inc., San Diego, CA USA

## Abstract

**Background:**

Pancreatic cancer is a recalcitrant disease in which R0 resection is often not achieved owing to difficulty in visualization of the tumor margins and proximity of adjacent vessels. To improve outcomes, we have developed fluorescence-guided surgery (FGS) and photoimmunotherapy (PIT) using a fluorescent tumor-specific antibody.

**Methods:**

Nude mice received surgical orthotopic implantation (SOI) of the human pancreatic cancer cell line BxPC-3 expressing green fluorescent protein. An anti-carcinoembryonic antigen-related cell adhesion molecule (CEACAM) monoclonal antibody (6G5j) was conjugated to the 700-nm fluorescent dye IR700DyeDX (6G5j-IR700DX). Three weeks after SOI, 16 mice received 50 μg 6G5j-IR700DX via the tail vein 24 h before surgery and were randomized to two groups: FGS-only (*n* = 8) and FGS + PIT (*n* = 8). All tumors were imaged with the Pearl Trilogy imaging system and resected under the guidance of the FLARE imaging system. The FGS + PIT group received PIT of the post-surgical bed at an intensity of 150 mW/cm^2^ for 30 min. Mice were sacrificed 4 weeks after initial surgery, and tumors were imaged with a Dino-Lite digital microscope, excised, and weighed.

**Results:**

The 6G5j-IR700DX dye illuminated the orthotopic pancreatic tumors for FGS and PIT. The metastatic recurrence rate was 100.0% for FGS-only and 25.0% for FGS + PIT (*p* = 0.007). The average total recurrent tumor weight was 2370.3 ± 1907.8 mg for FGS-only and 705.5 ± 1200.0 mg for FGS + PIT (*p* = 0.039).

**Conclusions:**

FGS and adjuvant PIT can be combined by using a single antibody–fluorophore conjugate to significantly reduce the frequency of pancreatic cancer recurrence.

Pancreatic cancer is a recalcitrant disease and one of the most fatal cancers with a 5-year survival rate of only about 9%.^1,2^ Even after potentially curative surgery, 80% of patients will suffer recurrence and die of the disease.^3^ This is due, in part, to the difficulty of visualizing surgical margins, the difficulty in performing extended resection owing to the proximity of adjacent blood vessels, especially in the uncinate process, and the presence of microscopic metastatic disease.

Fluorescence-guided surgery (FGS), which can use tumor-specific antibodies conjugated with fluorescent dyes to visualize primary tumors and metastases, is making progress in preclinical and clinical trials.^4,5^ In our laboratory, we have demonstrated the usefulness of FGS with an anti-carcinoembryonic antigen (CEA) antibody conjugated to a 650-nm fluorophore in a pancreatic cancer patient-derived orthotopic xenograft (PDOX) model.^6^ We have also shown the efficacy of a humanized anti-CEA antibody conjugated to an 800-nm fluorescent dye that labels pancreatic cancer in a PDOX mouse model.^7^

Photoimmunotherapy (PIT) using a target-specific photosensitizer based on a near-infrared (NIR) phthalocyanine dye conjugated to monoclonal antibodies has been developed for cancer therapy.^8^ PIT decreases the number of cancer cells and enhances host immune responses against the tumor. PIT has been used to treat local tumors as well as metastasis and prevents recurrence.^9^ In our laboratory, we have reported the usefulness of PIT with an anti-CEA antibody conjugated to 700-nm dye for pancreatic cancer nude mouse models.^10–12^ Initially, only PIT was used for the treatment of pancreatic cancer^10^ or bright-light surgery along with PIT.^11^

Carcinoembryonic antigen-related cell adhesion molecules (CEACAMs) are members of the carcinoembryonic antigen (CEA) gene family and the immunoglobulin superfamily.^13^ We have reported on the labeling of liver metastases from colorectal cancer using an anti-CEACAM antibody conjugated to a 700-nm fluorescent dye in a colon cancer liver metastases PDOX mouse model.^14^

The aim of the present study was to perform FGS and adjuvant PIT using a single antibody–fluorophore conjugate and to evaluate whether PIT can improve the prognosis of pancreatic cancer after FGS in an orthotopic mouse model.

## Methods

### Nude Mice

Nude (nu/nu) mice, aged 4–6 weeks, were purchased from Jackson Lab (Bar Harbor, ME). The animals were fed an autoclaved laboratory diet. All surgical procedures were performed under anesthesia by intramuscular injection of ketamine, xylazine, and acepromazine reconstituted in phosphate-buffered saline (PBS). Mice were treated with buprenorphine for pain control after surgical procedures. At the conclusion of the study, mice were euthanized with CO_2_ inhalation, which was confirmed with cervical dislocation. All studies were approved by the San Diego Veterans Administration Medical Center Institutional Animal Care and Use Committee (IACUC, animal use protocol A17-020).

### Cell Culture

Human pancreatic cancer cell line BxPC-3 expressing green fluorescent protein (GFP) (AntiCancer Inc., San Diego, CA) was maintained in Roswell Park Memorial Institute (RPMI) 1640 medium (GIBCO-BRL, Grand Island, NY). The medium was supplemented with 10% fetal calf serum (Hyclone, Logan, UT), 1% l-glutamine, and 1% penicillin/streptomycin (GIBCO-BRL). The cells were incubated at 37 °C in a 5% CO_2_ incubator.

### Cell Line Tumor

BxPC-3-GFP cells (1 × 10^6^) were initially injected subcutaneously in bilateral flanks of nude mice. Resulting tumors were harvested and sectioned into small pieces for surgical orthotopic implantation (SOI) to the pancreas.

### SOI of Pancreas Cancer to the Pancreas

The procedure of pancreatic cancer SOI was previously described.^15,16^ Briefly, a 10-mm transverse incision was made on the left flank of the mouse through the skin and peritoneum. The tail of the pancreas and spleen was exposed, and a single 1-mm^3^ tumor fragment from subcutaneous tumors was sutured to the tail of the pancreas using 8–0 surgical sutures (Ethicon Inc., Sommerville, NJ). The tail of the pancreas and spleen was returned to the abdomen, and the incision was closed with 6–0 surgical sutures (Ethicon Inc., Sommerville, NJ).

### Administration of Fluorescent Agents

Anti-CEACAM monoclonal antibody (6G5j) was a kind gift from Dr. B. B. Singer, Institute for Anatomy, Essen, Germany. The antibody was conjugated to a near-infrared dye IR700DyeDX NHS ester (LI-COR Biosciences Inc., Lincoln, NE) to establish 6G5j-IR700DX as per previously described methods.^14,17^

### Western Blotting

Tumor lysates were made using normal human pancreas tissue and the pancreatic cancer cell line BxPC-3-GFP xenograft. Western blotting was performed as previously described.^18^ Protein samples were isolated and transferred to Trans-Blot Turbo mini nitrocellulose membranes (Bio-Rad, Hercules, CA, Cat. 1704270) using the Bio-Rad Trans-Blot Turbo transfer system. The membrane was then incubated with the IRDye700DX-conjugated antibody at 4 °C overnight. The membrane was scanned with an Odyssey infrared imaging system (model 9120; LI-COR), and detection and quantification of band intensities were conducted using Image Studio Lite software (version 5.2; LI-COR). β-Actin was used as standard.

### Time Course of Fluorescence Intensity in Orthotopic Tumors

Three weeks after SOI, mice were administered 50 μg 6G5j-IR700DX via tail vein injection. In vivo imaging at 700-nm wavelength was performed daily up to 72 h using the Pearl Trilogy small animal imaging system (LI-COR). Image analysis was performed using Image Studio software small animal imaging analysis (LI-COR). The pancreas was set as the background, and an area of interest around the tumor fluorescence was automatically provided by the system, with a minimum of 250 pixels and at least 2.5 standard deviations from the background signal. The tumor-to-pancreas ratio (TPR) was calculated for each mouse by dividing the maximal fluorescence intensity (MFI) of the pancreatic tumor by the MFI of the adjacent pancreas.

### Intravital Static Imaging

Mice were anesthetized prior to imaging, and a laparotomy was performed to expose the pancreas. For 700-nm intravital static imaging, the Pearl Trilogy small animal fluorescence imaging system was used.

### FGS and PIT under Guidance of Intravital Dynamic Imaging

**Fig. 1:**
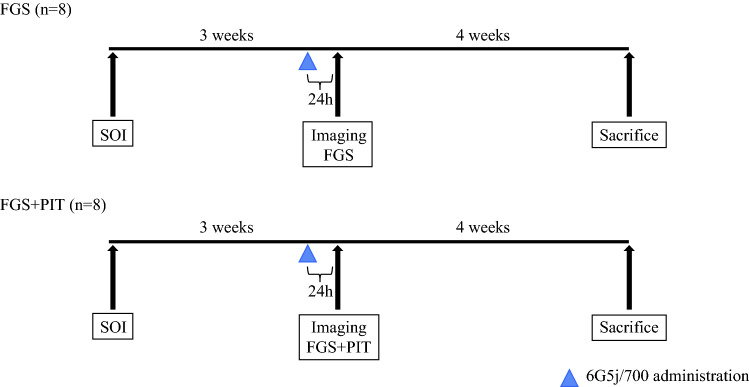
Schematic diagram of the experimental protocol. Surgical implantation of an BxPC-3-GFP tumor fragment in the tail of the pancreas was initially performed. Tumors grew 3 weeks, and mice were administered 50 μg 6G5j-IR700DX via tail vein injection 24 h before surgery. Sixteen mice were randomized to two groups: FGS-only and FGS + PIT. FGS was performed under guidance of the FLARE imaging system. In the FGS + PIT group, intraoperative PIT on the resection bed was performed with a 690-nm NIR laser for 30 min. Mice were sacrificed 4 weeks after initial surgery, and tumors were imaged with a Dino-Lite digital microscope, excised, and weighed

After confirmation of tumor engraftment, mice were randomized to two groups: FGS-only and FGS + PIT. Each treatment arm included eight tumor-bearing mice. A schematic diagram of the experimental protocol is shown in Fig. 1. Mice received 50 μg 6G5j-IR700DX via tail vein 24 h before surgery. For surgery, a 10-mm transverse incision was made on the left flank of the mouse through the skin and peritoneum and the tail of the pancreas and spleen was exposed through this incision. For intravital dynamic imaging, an overlay mode (bright light, 700 nm) with the FLARE imaging system (Curadel, MA) was used. A Dino-Lite digital microscope (AnMo Electronics Corporation, Taiwan) was also used to visualize GFP expression. FGS was performed after intravital static imaging. Manual pressure was applied to the resection site for hemostasis at the conclusion of the procedure.

In the FGS + PIT group, photoimmunotherapy was performed as described previously.^12,19^ The resection bed was then irradiated with light from a red light-emitting diode at 690-nm wavelength (NIR laser; Ultralasers, Inc., Newmarket, Ontario, Canada) and power density of 150 mW/cm^2^, as measured by an optical power meter (PM 100; Thorlabs, Inc., Newton, NJ) for 30 min. The distance from the laser source to the tumor was standardized at 15 cm for each mouse. Surrounding normal tissues were protected by aluminum foil during PIT.

After treatment completion, the remnant pancreas was gently returned to the peritoneal cavity, the abdominal wall and skin were closed with 6–0 surgical sutures (Ethicon Inc., Sommerville, NJ), and the mice were allowed to recover in their cages. Resected tumors were then sectioned from paraffin blocks, and hematoxylin and eosin (H&E) staining was performed at the University of California San Diego Moore’s Cancer Center Histology Core. Paraffin sections were imaged on a Lionheart FX microscope (Agilent Technologies Inc., Santa Clara, CA) to obtain bright-light images of H&E staining.

### Comparison of FGS-Only and FGS + PIT

To assess for postoperative recurrence, mice were sacrificed 4 weeks after initial surgery and underwent necropsy. Recurrent tumors expressing GFP were imaged with a Dino-Lite digital microscope, excised, and weighed in both groups. Local recurrence was defined as being located at the tail of the pancreas, where the tumor was resected, and having continuity with the pancreas, while other recurrences were defined as metastatic recurrence.

### Statistical Analysis

Statistical analysis was performed using SAS software (JMP 14.2.0; SAS Institute Inc., Cary, NC). Comparisons between categorical variables were analyzed with Fisher’s exact test. Continuous variables are expressed as mean ± standard deviation, and comparisons between continuous variables were determined with the Mann–Whitney *U* test. A *p*-value < 0.05 was considered statistically significant for all comparisons.

## Results

### Determination of CEACAM Expression

**Fig. 2:**
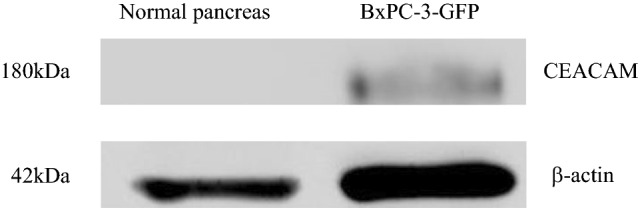
Western blot for CEACAM expression. BxPC-3-GFP expressed the CEACAM protein, whereas normal pancreas did not. β-Actin was used as standard

Western blotting was performed for normal pancreas and pancreatic cancer cell line BxPC-3-GFP, demonstrating the expression of CEACAM protein (Fig. 2). Normal pancreas did not express CEACAM protein.

### Tumor Labeling Time Course with 6G5j-IR700DX

**Fig. 3:**
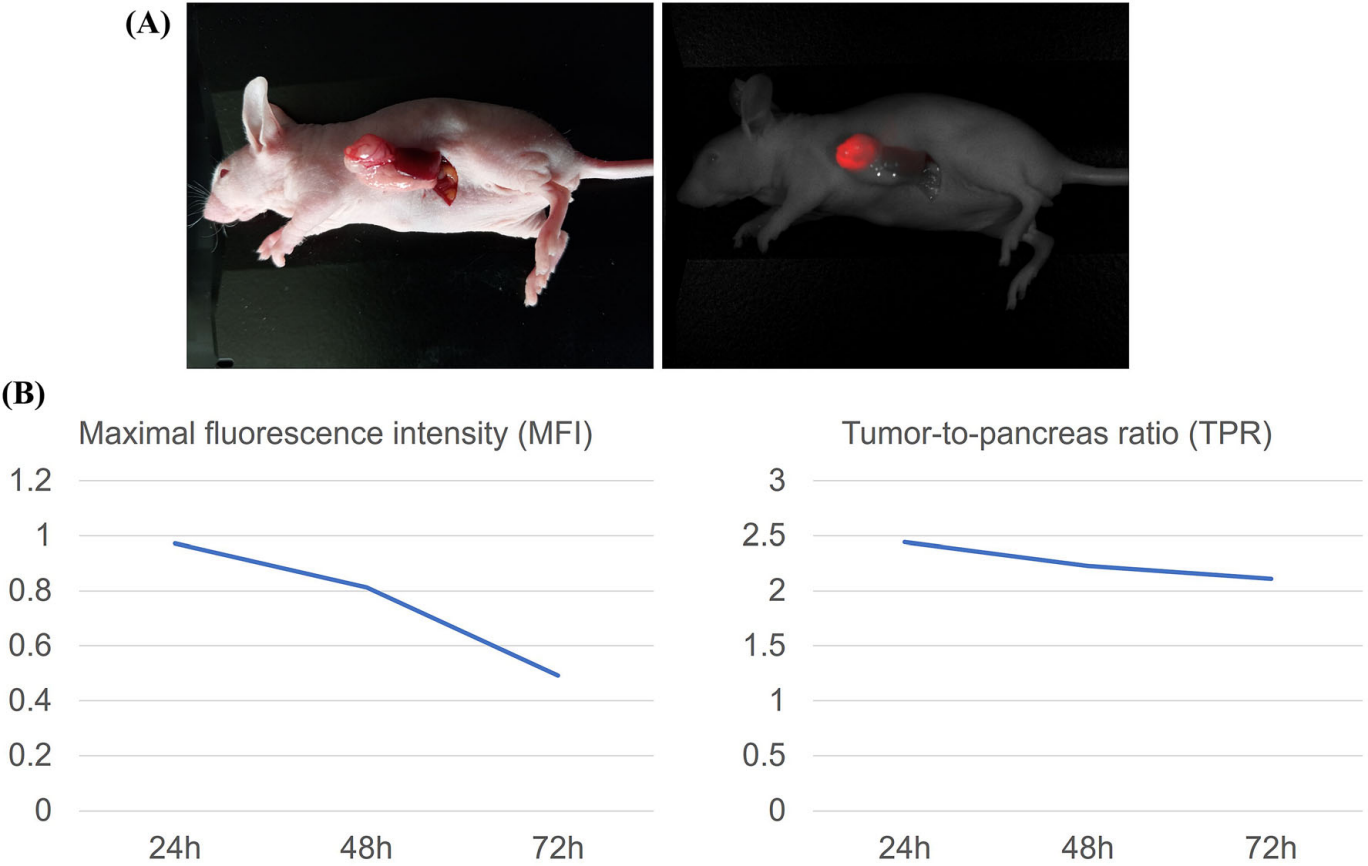
Time course imaging of orthotopic BxPC-3-GFP pancreatic cancer labeling with 6G5j-IR700DX. **A** Representative in vivo imaging of BxPC-3-GFP orthotopic pancreatic cancer model under bright field (left) and under 700-nm fluorescence (red pseudocolor) with the Pearl Trilogy small animal fluorescence imaging system (right). **B** Time course of maximal fluorescence intensity (MFI) and TPR, both decreasing monotonically with time

Intravital imaging of BxPC-3-GFP pancreatic cancer xenografts showed that tumors were visualized using 6G5j-IR700DX (Fig. 3A). Peak MFI and TPR was observed at 24 h (Fig. 3B).

### Labeling of Pancreatic Cancer for FGS and PIT

**Fig. 4:**
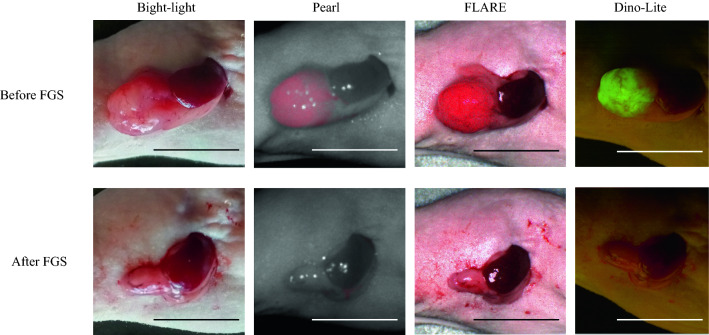
Labeling of orthotopic BxPC-3-GFP pancreatic cancer. 6G5j-IR700DX brightly illuminated the orthotopic pancreatic tumors visualized with the Pearl Trilogy small animal imaging system and the FLARE imaging system. GFP-expressing pancreatic cancers were visualized with the Dino-Lite digital microscope. BxPC-3-GFP pancreatic cancer before fluorescence guided surgery (FGS) and resection bed after FGS is shown, respectively. Scale bar 10 mm

The 6G5j-IR700DX brightly illuminated the orthotopic pancreatic tumors for FGS and PIT visualized with the Pearl Trilogy small animal imaging system and the FLARE imaging system. GFP-expression of the pancreatic cancer could also be detected with the Dino-Lite digital microscope (Fig. 4).

### FGS of the Pancreatic Cancer

**Fig. 5:**
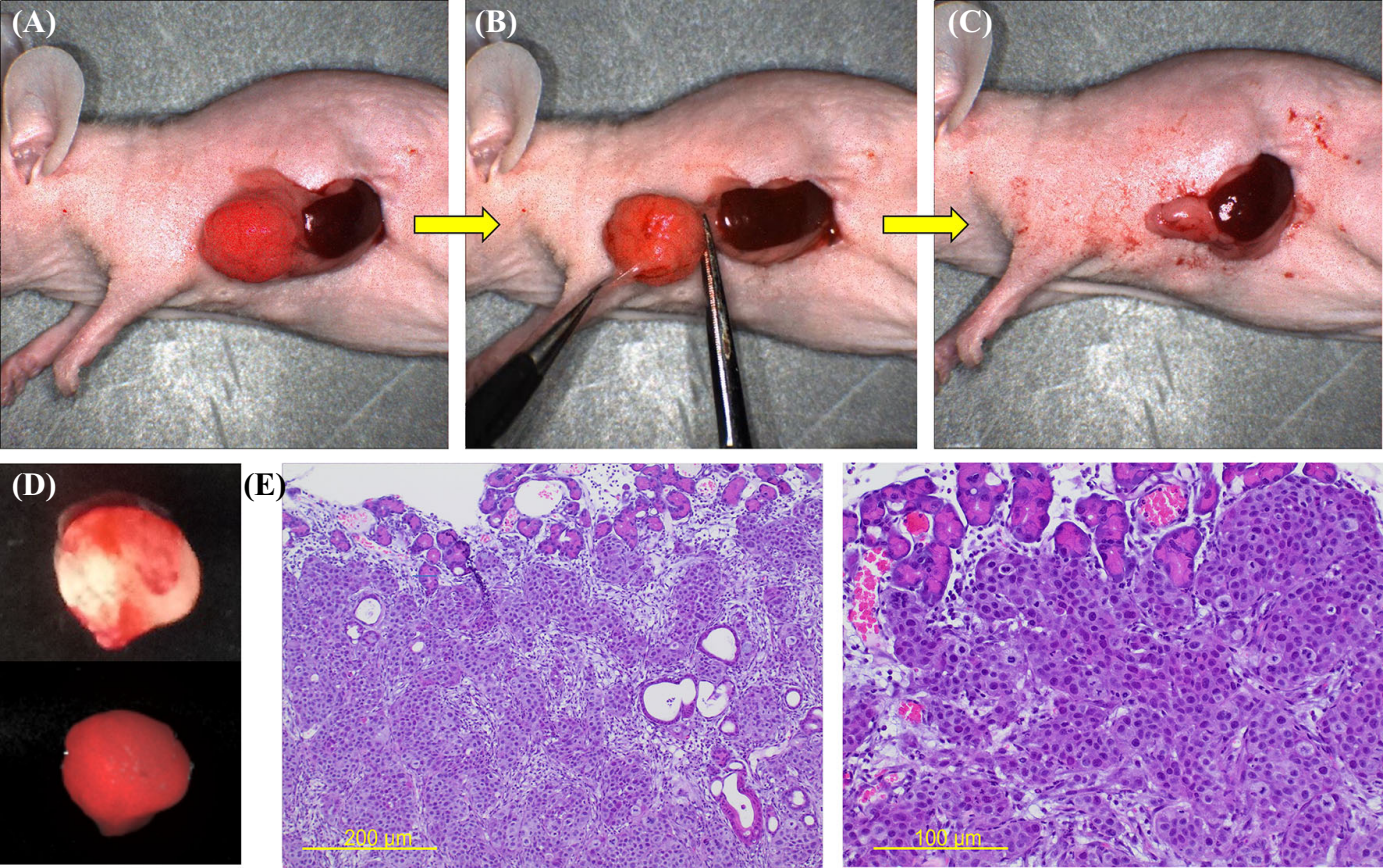
Fluorescence-guided surgery of BxPC-3-GFP orthotopic pancreatic cancer under guidance of the FLARE imaging system. The resection line was determined by the tumor margin on the basis of the fluorescence signal at 700 nm from 6G5j-IR700DX under the guidance of the FLARE imaging system. The pancreatic tumor appears red. **A** prior to FGS; **B** during FGS; **C** after FGS. **D** Representative bright-light and fluorescence images of the excised tumor; the volume and weight of the tumor is 175.0 mm^3^ and 266.7 mg, respectively. **E** The harvested pancreatic cancer after FGS is shown in hematoxylin and eosin-labeled sections, low-power (left, scale bar 200 µm) and high-power field (right, scale bar 100 µm)

The resection line was determined by the tumor margin on the basis of the fluorescence signal at 700 nm from 6G5j-IR700DX. The pancreatic tumor was resected with scissors, guided by fluorescence at all times (Fig. 5A–C). Grossly negative margins were obtained, and microscopic residual cancer was not visualized (Fig. 4). Representative bright-light and fluorescent images of the excised tumor are shown in Fig. 5D. The harvested pancreatic cancer was shown in H&E-labeled sections to have invaded the normal pancreas (Fig. 5E).

### Comparison of FGS-Only and FGS + PIT

**Fig. 6:**
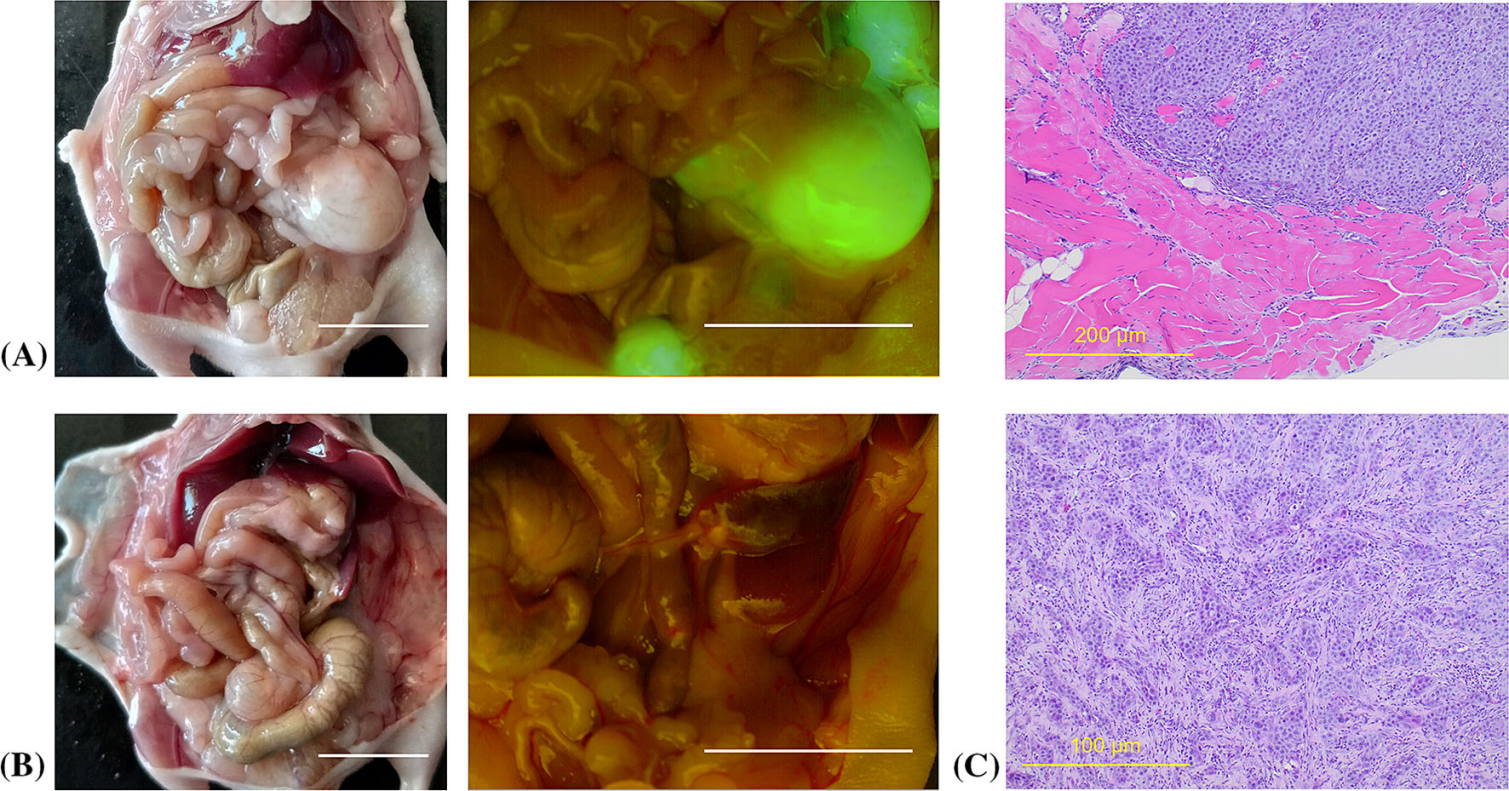
Postoperative recurrences from BxPC-3-GFP orthotopic pancreatic cancer 4 weeks after fluorescence guided surgery (FGS). **A** Representative images of recurrent tumors in FGS-only group; the tumor illuminated with GFP with the Dino-Lite digital microscope is a recurrence. **B** Representative images in the FGS + PIT group. No tumor was detected after FGS and PIT with the Dino-Lite digital microscope. **C** The harvested pancreatic cancer after FGS is seen in hematoxylin and eosin stained sections, low-power (upper, scale bar 200 µm) and high-power field (lower, scale bar 100 µm)

A representative image of recurrent tumors in the FGS-only group at 4 weeks after surgery is shown in Fig. 6A. The areas illuminated by GFP expression with the Dino-Lite digital microscope were resected as recurrent tumors. A representative image of the FGS + PIT group 4 weeks after surgery is shown in Fig. 6B. The harvested tumor showed invasion into the muscles and was mainly fibrostic with extensive stroma (Fig. 6C). A comparison of the perioperative data between the two groups is presented in Table 1. The local recurrence rate was 100.0% for the FGS-only group and 50.0% for the FGS + PIT group. The metastatic recurrence rate was 100.0% for the FGS-only group and 25.0% for the FGS + PIT group, being significantly less frequent in the latter. The total weight of recurrent tumors in the FGS +PIT group was significantly lower than that in the FGS group (2370.3 ± 1907.8 versus 705.5 ± 1200.0 mg, *p* = 0.039).

**Table 1:** Comparison of perioperative outcomes between FGS-only and FGS combined with PIT

	FGS-only (*n* = 8)	FGS + PIT (*n* = 8)	*p*-value
Body weight (g)	26.4 ± 3.0	26.8 ± 2.8	0.65
Tumor-to-pancreas ratio	3.60 ± 0.38	3.83 ± 0.83	0.51
Tumor volume (mm^3^)	211.3 ± 48.4	207.3 ± 95.6	0.57
Specimen weight (mg)	316 ± 109	260 ± 170	0.19
Local recurrence rate (%)	100.0%	50.0%	0.077
Metastatic recurrence rate (%)	100.0%	25.0%	0.0070
Total recurrent tumor weight (mg)	2370.3 ± 1907.8	705.5 ± 1200.0	0.039

## Discussion

In the present study, we demonstrated that the combination of FGS and adjuvant PIT, using a single anti-CEACAM antibody with a 700-nm fluorophore conjugate, could reduce recurrences in an orthotopic mouse model of pancreatic cancer.

Medical devices for FGS used in clinical practice are mainly 800 nm for ICG. Our laboratory has reported colon or pancreatic cancer labeling using tumor-specific antibodies conjugated to a 800-nm fluorescent dye.^7,20,21^ Anti-CEACAM has been shown to be useful for detection of colon cancer at both 800 and 700 nm in mouse models.^14,17^ Although 700 nm is less penetrant than 800 nm, the development of medical devices for FGS using 700 nm has been progressing. The introduction of FGS using a 700-nm fluorescent dye is promising for the advancement of color-coded surgery, in which organs are fluorescently labeled at various wavelengths.

PIT is a molecularly targeted phototherapy for cancer that is based on injecting a conjugate of a near-infrared IR700DX and a monoclonal antibody that targets an antigen expressed on the cancer cell surface. Subsequent local exposure to NIR light results in rapid and highly selective immunogenic death of targeted cancer cells.^9^ NIR-PIT not only treats the targeted local tumor but also reduces or eliminates systemic metastasis and prevents recurrence in animal models.^22, 23^ In the present study, PIT performed as an intraoperative adjuvant therapy reduced postoperative recurrence in an orthotopic mouse model of pancreatic cancer.

Our previous report clarified that 6G5j can bind to CEACAM1, CEACAM3, CEACAM5 (conventional CEA), CEACAM6, and CEACAM8.^17^ The ability to bind to multiple CEACAMs may improve the binding capacity of the antibody and enhance the visualization of tumor margins that are otherwise invisible compared with CEA alone.

Regarding the association between CEACAM and pancreatic cancer, approximately 70% of all analyzed tumor tissues showed expression of CEACAM 1, 5, or 6, or a combination of all of them.^24^ CEACAM 5 and 6 expression were correlated with lymph node metastasis, and patients with high expression of CEACAM 5 or 6 had shorter overall survival and disease-free survival.^24^ A CEACAM antibody is expected to be useful in the targeting of pancreatic cancer, and the present study demonstrated this in a BxPC-3-GFP pancreatic cancer mouse model.

This is the first report of combined FGS and PIT using a single fluorescent antibody. If multiple agents are used in combination, there could be concern about their interactions; however, a single compound could reduce the likelihood of this happening.

The present proof-of-principle study demonstrates that FGS and intraoperative adjuvant PIT can be combined by using 6G5j-IR700DX in an orthotopic mouse model of the human BxPC-3-GFP pancreatic cancer cell line with the combination contributing to the inhibition of tumor recurrence. Currently, pancreatic cancer located in the uncinate process has a higher rate of R1 resection than nonuncinate process pancreatic head cancers. In addition, survival rates are lower after R0 resection of uncinate process pancreatic cancer.^25^ To improve outcomes in this recalcitrant cancer, we will first develop, in future experiments, an orthotopic model of uncinate cancer to determine whether FGS can improve outcomes. Secondly, we will develop the orthotopic uncinate process model in humanized NSG mice engrafted with peripheral blood lymphocytes.^26^ We will combine FGS and photoimmunotherapy (PIT) on the orthotopic uncinate process cancer in the humanized model to determine the advantage of the combined modalities.

## Conclusions

FGS and adjuvant PIT can be combined by using a single anti-CEACAM antibody with 700-nm fluorophore conjugate in an orthotopic mouse model of pancreatic cancer. Adjuvant intraoperative PIT after FGS could significantly reduce the frequency of recurrence and the total weight of recurrent tumors, which suggests clinical potential of combining FGS with PIT.
